# Four Synthetic Cathinones: 3-Chloromethcathinone, 4-Chloromethcathinone, 4-Fluoro-α-Pyrrolidinopentiophenone, and 4-Methoxy-α-Pyrrolidinopentiophenone Produce Changes in the Spontaneous Locomotor Activity and Motor Performance in Mice with Varied Profiles

**DOI:** 10.1007/s12640-020-00227-8

**Published:** 2020-06-06

**Authors:** Jakub Wojcieszak, Katarzyna Kuczyńska, Jolanta B. Zawilska

**Affiliations:** Department of Pharmacodynamics, Medical University of Lodz, 90-151 Lodz, Poland

**Keywords:** Spontaneous locomotor activity, Rotarod, 4-CMC, 3-CMC, 4-MeO-PVP, 4-F-PVP

## Abstract

Two chloromethcathinones, 3-chloromethcathinone (3-CMC) and 4-chloromethcathinone (4-CMC), and two *para*-substituted α-pyrrolidinophenones, 4-methoxy-α-pyrrolidinopentiophenone (4-MeO-PVP) and 4-fluoro-α-pyrrolidinopentiophenone (4-F-PVP), represent synthetic cathinones, the second most frequently abused group of new psychoactive substances (NPSs), which has aroused a worldwide health concern in the last decade. Synthetic cathinones act as psychostimulants by elevating extracellular levels of monoaminergic neurotransmitters. This study investigates effects of 3-CMC, 4-CMC, 4-MeO-PVP, and 4-F-PVP on the spontaneous locomotor activity and motor performance of mice. Additionally, neurotoxicity of substituted methcathinones against SH-SY5Y neuroblastoma cells was evaluated. All test cathinones stimulate in a dose-dependent manner horizontal locomotor activity of mice. Consistently to our prior findings, pyrrovalerones, but not methcathinone derivatives, produce dose-dependent elevation of vertical locomotor activity (rearing behavior). None of the tested compounds decreases the time spent on the accelerating rotarod, pointing to the lack of considerable motor disability in mice after acute exposition. Only 4-MeO-PVP at the high tested dose (20 mg/kg) increases motor performance of mice. Considering that α-pyrrolidinophenones are highly potent and selective DA uptake inhibitors, while chloromethcathinones enhance non-selective DA/5-HT release, we suggest that the increase of vertical locomotor activity and performance on rotarod in mice may serve as a behavioral indicator of the monoaminergic profile of synthetic cathinones. Finally, this study gives first insights into cytotoxicity of both 3-CMC and 4-CMC displayed against SH-SY5Y cells, which emerges and intensifies after prolonged incubation, suggesting the indirect mechanism of action, unrelated to interactions with monoamine transporters.

## Introduction

New psychoactive substances (NPSs) are a heterogeneous and rapidly developing group of recreational drugs, posing a serious threat to public health (EMCDDA 2017, 2018, 2019). Synthetic cathinones, a subgroup of NPSs, are analogs of (−)-cathinone, which is a naturally occurring psychostimulating phenylalkylamine alkaloid present in fresh leaves of khat shrub (*Catha edulis*) (Feng et al. 2017; Simmons et al. 2018). Synthetic cathinones appeared on the drug market in the mid-2000s as an alternative to scheduled psychostimulants. Products containing these compounds are usually mislabeled as “not for human consumption” to circumvent legal control actions. Once a particular constituent of “bath salts” is outlawed, new compounds with slightly modified chemical structures emerge, making synthetic cathinones the second largest group of NPSs monitored by the European Monitoring Centre for Drug and Drug Addiction (EMCDDA) (Coppola and Mondola 2012; EMCDDA 2019; Zawilska and Wojcieszak 2013). Chlorine-containing derivatives have become one of the most popular synthetic cathinones after the appearance of 4-chloromethcathinone (4-CMC; clephedrone) around 2014 (Taschwer et al. 2014; Wiergowski et al. 2017). Halogenated methcathinones, namely 4-CMC and 3-chloromethcathinone (3-CMC), were among the five most frequently seized cathinones in Europe in 2016 (Białas et al. 2017; EMCDDA 2018). During that time, diverse α-pyrrolidinophenone (pyrovalerone) derivatives of cathinone emerged on the market when 3,4-methylenedioxypyrovalerone (3,4-MDPV) was outlawed (Zawilska and Wojcieszak 2017). Substitution of the phenyl ring in the *para*-position was one of the most common modifications of pyrovalerones, giving rise to new compounds, such as 4-fluoro-α-pyrrolidinopentiophenone (4-F-PVP) or 4-methoxy-α-pyrrolidinopentiophenone (4-MeO-PVP), which were originally detected in Japan in 2013, and then in Germany (Ellefsen et al. 2016; Zawilska and Wojcieszak 2017).

Synthetic cathinones exert similar effects to psychostimulants like methamphetamine and cocaine or to an emphatogen MDMA (Eshleman et al. 2017). Typical desired effects evoked by them include increased alertness and awareness, increased energy and motivation, euphoria, excitement, improved mood and mild empathogenic effects like openness in communication, sociability and talkativeness, intensification of sensory experiences, music sensitivity, and moderate sexual arousal. Synthetic cathinones also induce a wide range of toxic effects on numerous body systems. Acute intoxication is primarily manifested by neurological, cardiovascular, and psychopathological symptoms; the most prominent are the following: tachycardia, hypertension, chest pain, hyperthermia, insomnia, agitation, hallucinations, delusions, and confusion (Coppola and Mondola 2012; Zawilska and Wojcieszak 2013).

Synthetic cathinones elicit their pharmacological effects by elevating extracellular levels of monoamine neurotransmitters. A chemical structure of compounds determines their selectivity for particular monoamine transporting proteins, dopamine transporter (DAT), serotonin transporter (SERT), and noradrenaline transporter (NET), and whether they are monoamine reuptake inhibitors or monoamine releasers (Eshleman et al. 2017; Simmler et al. 2013).

Pyrovalerone cathinones are very potent and selective monoamine reuptake inhibitors. In general, they demonstrate high affinity for DAT and NET, whereas their affinity for SERT is negligible (Zawilska and Wojcieszak 2017). Significantly higher selectivity for DAT over SERT indicates that α-pyrrolidinophenones may have very high abuse potential (Eshleman et al. 2017; Zawilska and Wojcieszak 2017). It has been demonstrated that they are potent psychostimulants producing locomotor activation mediated by stimulation of D_1_-dopamine receptors (Wojcieszak et al. 2018b; Zawilska and Wojcieszak 2017). High potency of pyrovalerones is also related to their pharmacokinetic properties, as the pyrrolidine ring, determining high lipophilicity, enables a rapid and effective permeation of the compound through the blood-brain barrier. Because of insignificant SERT inhibition, pyrovalerone derivatives neither have entactogen properties nor raise the body temperature (Zawilska and Wojcieszak 2017).

Substituted methcathinones bear a structural resemblance to methamphetamine and MDMA (Eshleman et al. 2013). These compounds may play a role of monoamine transporter inhibitors like butylone, or substrates like 4-fluoromethcathinone (4-FMC), mephedrone, methylone, or 4-CMC (Eshleman et al. 2013, 2017). A steric bulk of the *para* substituent is a key factor of selectivity for monoamine transporters. Compounds with minor steric bulk display higher affinity for DAT, whereas compounds with greater steric bulk exhibit higher selectivity for SERT (Bonano et al. 2015). Methcathinone derivatives with equivalent affinity for DAT and SERT or higher affinity for SERT over DAT show empathogenic properties. They induce moderate increase of locomotor activity and are endowed with a lower abuse potential compared with pyrovalerones (Bonano et al. 2015; Eshleman et al. 2017).

Toxicity of synthetic cathinones seems to be similar to harmful effects of amphetamine and MDMA (den Hollander et al. 2015). One of prime reasons of neurotoxicity may be an increasing neuronal oxidative stress from reactive oxygen and nitrogen species (den Hollander et al. 2015; Matsunaga et al. 2017; Valente et al. 2017) as well as depletion of reduced glutathione, which result in impairing mitochondrial functions and lead to cell apoptosis (Matsunaga et al. 2017; Valente et al. 2017).

Although synthetic cathinones have gained great popularity recently, data on their pharmacological activity and toxicity is very limited. Knowledge on desired and side effects of these compounds is largely based on personal experience of abusers, as described on Internet forums, or from published case reports of patients admitted to hospitals due to acute intoxications (Taschwer et al. 2014).

The aim of this study is to assess in vivo pharmacological activity of four widely abused synthetic cathinones from two groups:

- Substituted methcathinones: 3-chloromethcathinone (3-CMC) and 4-chloromethcathinone (4-CMC).
- Substituted α-pyrrolidinophenones: 4-methoxy-α-pyrrolidinopentiophenone (4-MeO-PVP) and 4-fluoro-α-pyrrolidinopentiophenone (4-F-PVP).

Additionally, in vitro neurotoxicity of substituted methcathinones was evaluated.

Pharmacological activity was assessed in mice by measuring changes in spontaneous locomotor activity as a marker of psychostimulant properties. Neurotoxicity of methcathinones was evaluated in vitro by measuring viability of SH-SY5Y neuroblastoma cells with MTT and LDH tests. Performance of mice on an accelerating rotarod was conducted in order to assess an effect on the forced locomotor activity, which is related to psychostimulant properties of drugs, or to detect the eventual impairment of motor coordination, which is a behavioral marker of cerebellar dopaminergic dysfunctions in rodents (Giannotti et al. 2017; Shiotsuki et al. 2010).

## Materials and Methods

### Reagents

Synthetic β-cathinones: 3-chloromethcathinone [3-CMC, 1-(3-chlorophenyl)-2-(methylamino)-1-propanone], 4-chloromethcathinone [4-CMC, 1-(4-chlorophenyl)-2-(methylamino)-1-propanone], 4-methoxy-α-pyrrolidinopentiophenone [4-MeO-PVP, 1-(4-methoxyphenyl)-2-(1-pyrrolidinyl)-1-pentanone], and 4-fluoro-α-pyrrolidinopentiophenone [4-F-PVP, 1-(4-fluorophenyl)-2-(1-pyrrolidinyl)-1-pentanone] were purchased in the form of their hydrochloride salts from Cayman Chemical (Ann Arbor, MI, USA). Isotonic solution of saline for injections (0.9% NaCl) was purchased from Polska Grupa Farmaceutyczna (Łódź, Poland). Cell culture reagents: Dulbecco’s modified Eagle’s medium with F12 supplement (DMEM/F12), heat-inactivated fetal bovine serum (FBS), phosphate-buffered saline (PBS), Trypsin-EDTA, penicillin, streptomycin, and amphotericin B were purchased from Life Technologies (Warsaw, Poland). Dimethyl sulfoxide (DMSO), Triton X-100, and MTT (3-(4,5-dimethyl-2-thiazolyl)-2,5-diphenyl-2*H*-tetrazolium bromide) were purchased from Sigma-Aldrich (Poznań, Poland).

### Cell Culture

Human neuroblastoma SH-SY5Y (ATCC® CRL-2266™) cell line, purchased from Leibniz Institute DSMZ-German Collection of Microorganisms and Cell Cultures (DSMZ, Braunschweig, Germany), was cultured in DMEM/F12 with 10% FBS, penicillin (100 U/mL), streptomycin (100 μg/mL), and amphotericin B (0.25 μg/mL) at 37 °C in a humidified atmosphere enriched with 5% CO_2_. Upon reaching 80–90% confluency, cells were harvested with 0.25% trypsin in 1 mM EDTA for 3 min and transferred into 96-well microplates for cell viability experiments.

### MTT Assay

MTT assay was performed as previously described (Wojcieszak et al. 2018a). After overnight incubation of cells in 96-well microplate at the density of approx. 10,000 cells/well, the complete culture medium was removed and replaced by working solutions of the tested compounds prepared in FBS-free medium. Fresh medium without FBS was used for the control group.

Cell viability and mitochondrial function were measured by assessment of MTT [3-(4,5-dimethyl-2-thiazolyl)-2,5-diphenyl-2*H*-tetrazoliumbromide] reduction by mitochondrial dehydrogenases after 24- and 72-h exposure to the tested drugs. A solution of MTT (1.25 mg/mL) was added to the cells, and the culture was incubated for a further 3 h at 37 °C. After aspiration of culture medium, formazan crystals were dissolved in DMSO, and its absorbance was measured at 570 nm using Bio-Rad microplate reader model 680, this value being proportional to the number of cells with intact mitochondria. As the tested methcathinones express ability to reduce MTT nonenzymatically, all experiments were paralleled with blanks containing solutions of the drugs in culture medium and MTT without cells. The mean optic density (OD) values for each treatment group were calculated by subtraction of the blank value from the value of corresponding treated cells. The results are expressed as percentages of the control group values, being considered 100% viable.

### LDH Assay

LDH assay, based on the measurement of the activity of lactate dehydrogenase released from damaged cells into the medium, was performed to assess cell membrane integrity. SH-SY5Y cells (approx. 20,000 cells/well) were treated with 3-CMC and 4-CMC dissolved in a serum-free medium without phenol red for 48 h. Negative control group was treated with the medium, while positive control was treated with 1% *v*/*v* TritonX-100. Measurement of LDH activity was conducted using LDH Cytotoxicity Assay (ScienCell Research Laboratories, Carlsbad, CA, USA) according to the manufacturer’s instructions. Results are expressed as the percent of positive control group, considered as 100% cytotoxicity.

### Animals

Experiments were performed on adult male C57BL/6J inbred mice. All housing conditions and experimental procedures were in accordance with the European Union guidelines regarding the care and use of laboratory animals (European Communities Council Directive of September 2010 (2010/63/EU)), and were approved by the Local Ethical Commission for Experimentations on Animals in Łódź. The animals were housed in a sound-attenuated chambers, four per cage, with automatic 12-h light/dark cycles (light phase beginning at 6:00 a.m.), with free access to drinking water and standard food. All experiments were performed during the light cycle (8:00–14:00). The mice were at 9 weeks of age as the experiment began with an assessment of spontaneous locomotor activity. After 2 weeks of washout, the groups were counterbalanced and a rotarod test was performed.

### Locomotor Activity

The procedure was conducted as formerly described, with slight modifications (Wojcieszak et al. 2018b). The spontaneous locomotor activity was measured using Opto-Varimex Auto-Track (model 0271-002M, Columbus Instruments, Columbus, OH, USA) open field chambers (20.3 × 20.3 × 20.3 cm) in set of four. Each chamber was equipped with infrared beams (16 beams) and corresponding photodetectors, spaced by 1.3 cm, located on the X and Y horizontal axes. Additionally, identical sets of infrared emitters and detectors were installed on the higher layer in order to detect vertical movements (rearing behavior).

Mice were randomly assigned to treatment groups, each consisting of eight animals: 3-CMC (5, 10, 20 mg/kg), 4-CMC (5, 10, 20 mg/kg), 4-MeO-PVP (5, 10, 20 mg/kg) or 4-F-PVP (5, 10, 20 mg/kg) and a control group. Drug solutions prepared in 0.9% NaCl or 0.9% NaCl solution for the control group were injected subcutaneously (s.c.) in a volume of 0.1 mL/10 g of body mass immediately before the start of the experiment. Experimental sessions lasted for 120 min and were conducted in a sound-attenuated room with a dim red light (invisible for rodents) from above. Experimental analysis was based on the counts of beam breaks on the bottom and top layers within 10-min intervals.

### Motor Performance

A motor performance of mice was evaluated in an accelerated rotarod test (Rotarod, model RTD-4, Ataner, Lublin, Poland) by measuring latency to fall off a revolving rod. Mice were placed on a horizontal rotating bar (3 cm in diameter, 5.5 cm in width) made of non-slippery, sandy polyvinyl chloride not permitting the animals to grip it (Shiotsuki et al. 2010). The start speed was set on 4 rpm for the first 10 s of the experiment; then, the acceleration rate was adjusted to 20 rpm/min with the maximum speed of 40 rpm (Deacon 2013). The running time was continuously recorded, and it stopped automatically when a mouse fell off the bar. After the fall the mouse was returned to its home cage to rest. To avoid affecting results by the aspect of learning, and to obtain not statistically different basal performance of mice across all groups, animals were trained for three consecutive days prior to the test day. A training phase consisted of three trials per day, 5 min apart. On the test day, mice were injected s.c. with vehicle (0.9% saline), 3-CMC (10 and 20 mg/kg), 4-CMC (10 and 20 mg/kg), 4-MeO-PVP (10 and 20 mg/kg), or 4-F-PVP (10 and 20 mg/kg). The assessment of motor coordination started 10 min after injection. Each mouse was subjected to four trials according to the same procedure as on the training days.

### Data Analysis

Statistical analysis was performed using GraphPad Prism 6.0 software (GraphPad, San Diego, CA, USA). The results were recognized as statistically significant when *p* < 0.05.

### Cytotoxicity Assays

All analyses were performed on the data obtained from at least three independent experiments, each done in six replicates. Effects of 3-CMC and 4-CMC on cell viability were analyzed using one-way ANOVA, when eligible followed with Dunnett’s post hoc test to compare treated groups with a proper control in the case of normal distribution (MTT) or Kruskal-Wallis test, when eligible followed by Dunn’s post hoc test in the case of non-Gaussian distribution (LDH).

### Spontaneous Locomotor Activity

Locomotor activity was expressed as the total distance traveled (cm) and total number of rearings during each 10-min bins during the 120-min experiments. For time course analysis, two-way repeated measures ANOVA (time, treatment) was conducted followed by Dunnett’s post hoc test for multiple comparisons. Additionally, total effects during 120 min of experiment were analyzed using one-way ANOVA followed by Tukey’s post hoc test.

### Rotarod

Values of time spent on the rotarod on day 3 (last training day) and day 4 (test day) were analyzed using one-way ANOVA. In the case of *p* < 0.05 (4-MeO-PVP day 4), Tukey’s post hoc test was conducted for multiple comparisons.

## Results

### Spontaneous Locomotor Activity

**Fig. 1:**
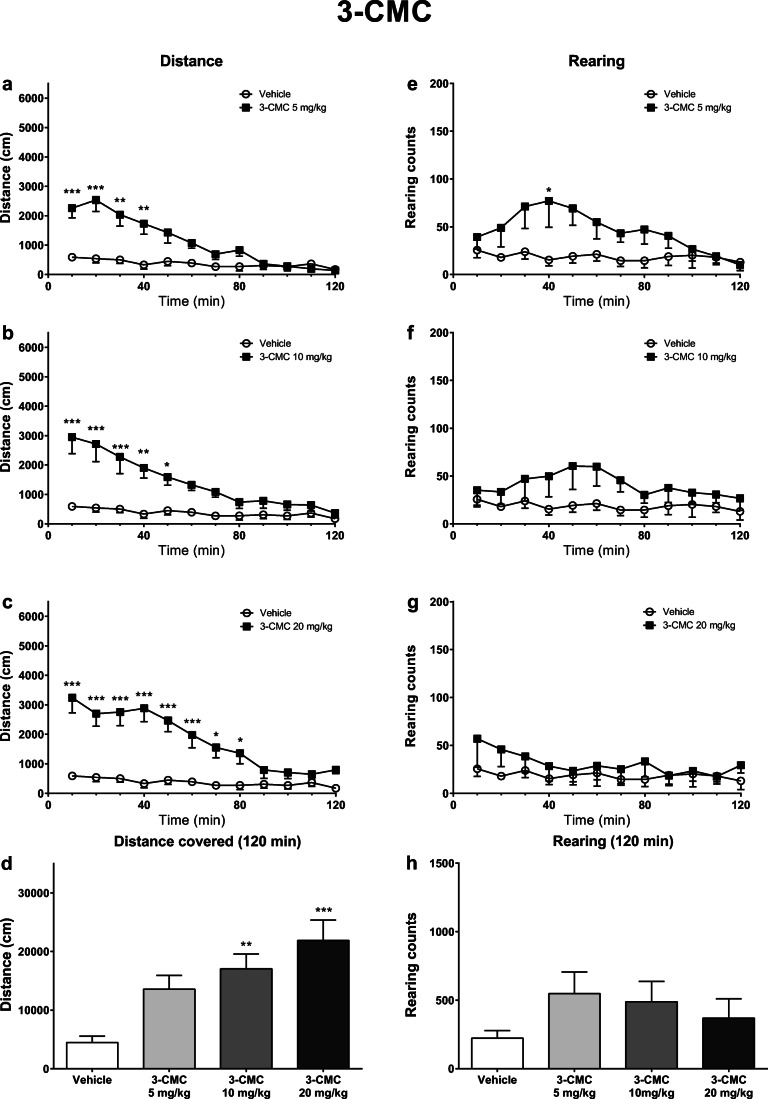
Effects of 3-CMC (5, 10, 20 mg/kg) on the spontaneous locomotor activity of mice. Average horizontal (**a**–**c**) and vertical (**e**–**g**) activities in 10-min bins. Total distance traveled during 120 min (**d**). Total rearing counts during 120 min (**h**). Data presented as mean ± standard error of the mean (SEM) (*n* = 8). ***p < 0.001; **p < 0.01; *p < 0.05 vs. control group

**Fig. 2:**
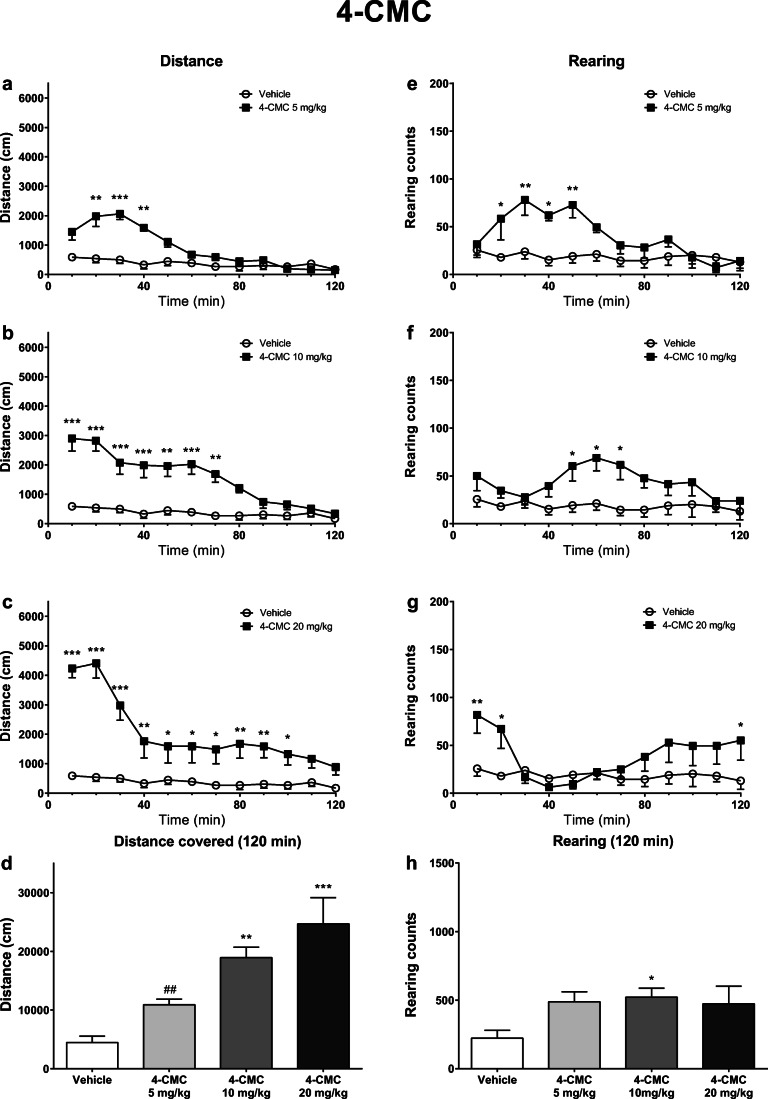
Effects of 4-CMC (5, 10, 20 mg/kg) on the spontaneous locomotor activity of mice. Average horizontal (**a**–**c**) and vertical (**e**–**g**) activities in 10-min bins. Total distance traveled during 120 min (**d**). Total rearing counts during 120 min (**h**). Data presented as mean ± standard error of the mean (SEM) (*n* = 8). ***p < 0.001; **p < 0.01; *p < 0.05 vs. control group; ##*p* < 0.01 vs. 4-CMC 20 mg/kg group

**Fig. 3:**
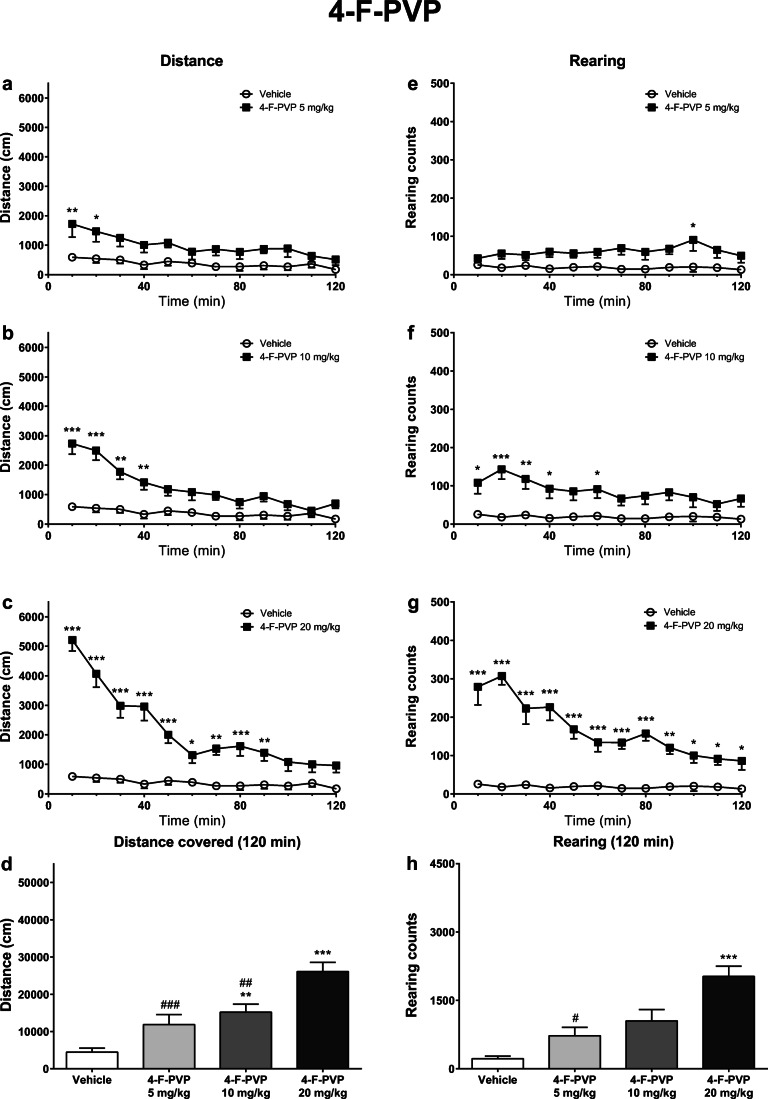
Effects of 4-F-PVP (5, 10, 20 mg/kg) on the spontaneous locomotor activity of mice. Average horizontal (**a**–**c**) and vertical (**e**–**g**) activities in 10-min bins. Total distance traveled during 120 min (**d**). Total rearing counts during 120 min (**h**). Data presented as mean ± standard error of the mean (SEM) (*n* = 8). ***p < 0.001; **p < 0.01; *p < 0.05 vs. control group; ###*p* < 0.001; ##*p* < 0.01; #*p* < 0.05 vs. 4-F-PVP 20 mg/kg group

**Fig. 4:**
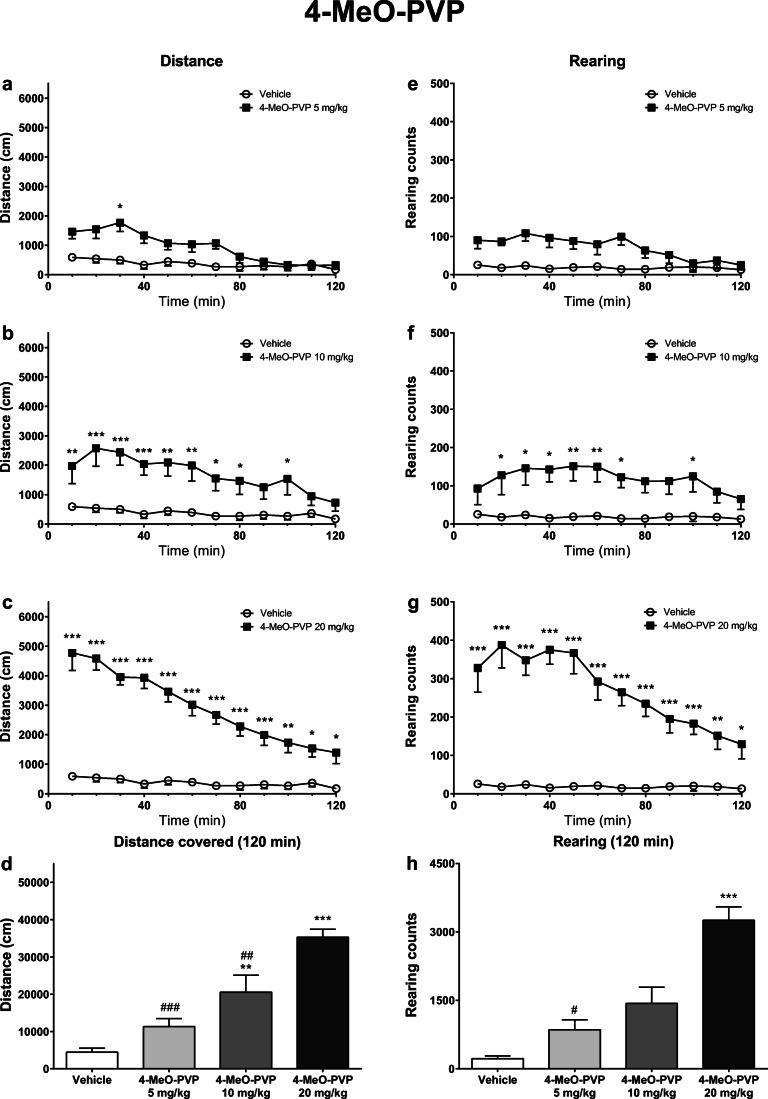
Effects of 4-MeO-PVP (5, 10, 20 mg/kg) on the spontaneous locomotor activity of mice. Average horizontal (**a**–**c**) and vertical (**e**–**g**) activities in 10-min bins. Total distance traveled during 120 min (**d**). Total rearing counts during 120 min (**h**). Data presented as mean ± standard error of the mean (SEM) (*n* = 8). ***p < 0.001; **p < 0.01; *p < 0.05 vs. control group; ###*p* < 0.001; ##*p* < 0.01; #*p* < 0.05 vs. 4-MeO-PVP 20 mg/kg group

All tested compounds caused time- and dose-dependent increases of horizontal spontaneous locomotor activity in mice, while only α-pyrrolidinophenones (4-F-PVP and 4-MeO-PVP), but not chloromethcathinones, increased vertical spontaneous locomotor activity in a dose-dependent manner (Figs. 1, 2, 3, and 4).

In mice treated with 3-CMC, horizontal activity was significantly affected by the treatment (*F*_3,28_ = 8.549; *p* = 0.0003), time (*F*_11,308_ = 40.91; *p* < 0.0001), and treatment × time interaction (*F*_33,308_ = 3.835; *p* < 0.0001). Within 10-min bins, a significant elevation of locomotor activity vs. control group was observed 0–40 min (5 mg/kg), 0–50 min (10 mg/kg), and 0–80 min (20 mg/kg) post-injection intervals (Fig. 1a–c). Additional analysis demonstrated that a total distance traveled within 120 min of the experiment was significantly higher compared with control in mice receiving 3-CMC at 10 mg/kg and 20 mg/kg (Fig. 1d).

Vertical locomotor activity in mice treated with 3-CMC was significantly affected only by the time (*F*_11,308_ = 2.917; *p* = 0.0011), but not by treatment (*F*_3,28_ = 1.175; *p* = 0.3370) or treatment × time interaction (*F*_33,308_ = 1.290; *p* = 0.1388). However, within 10-min time bins, vertical locomotor activity, expressed as the number of rearings, was increased compared with control only after the lowest tested dose of 5 mg/kg during 30–40 min post-injection interval (Fig. 1e–g). 3-CMC did not produce significant changes in the total vertical activity during 120 min of experiment (Fig. 1h).

Administration of 4-CMC to mice led to a potent stimulation of horizontal locomotor activity, which was significantly influenced by the treatment (*F*_3,28_ = 12.53; *p* < 0.0001), time (*F*_11,308_ = 38.16; *p* < 0.0001), and time × treatment interaction (*F*_33,308_ = 5.788; *p* < 0.0001). Within 10-min bins, horizontal activity was increased during 10–40 min post-injection interval after 4-CMC at 5 mg/kg, 0–70 min post-injection at 10 mg/kg, and 0–100 min post-injection at 20 mg/kg (Fig. 2a–c). A total distance traveled during 120 min was significantly higher compared with the control group after treatment with 4-CMC at doses of 10 mg/kg and 20 mg/kg. Mice treated with 20 mg/kg 4-CMC also traveled a longer distance compared with the 5 mg/kg group (Fig. 2d).

After treatment with 4-CMC, vertical locomotor activity of mice was significantly affected by the time (*F*_11,308_ = 1.836; *p* = 0.0475) and treatment × time interaction (*F*_33,308_ = 3.721; *p* < 0.0001), but not treatment itself (*F*_3,28_ = 2.596; *p* = 0.0722). Within 10-min bins, 4-CMC significantly elevated vertical activity in 10–50 min (5 mg/kg), 40–70 min (10 mg/kg), 0–20 and 110–120 min (20 mg/kg) post-injection intervals (Fig. 2e–g). A total number of rearings was significantly higher compared with the control group only after treatment with 10 mg/kg of 4-CMC, with no upward trend or inverted U-shaped curve (Fig. 2h).

Treatment of mice with 4-F-PVP resulted in an increase of their horizontal locomotor activity, which was significantly dependent on the treatment (*F*_3,28_ = 16.90; *p* < 0.0001), time (*F*_11,308_ = 45.54; *p* < 0.0001), and treatment × time interaction (*F*_33,308_ = 8.937; *p* < 0.0001). Significant elevation of locomotor activity vs. control group was observed in 0–20 min (5 mg/kg), 0–40 min (10 mg/kg), and 0–90 min (20 mg/kg) post-injection intervals (Fig. 3a–c). A total distance traveled during 120 min was significantly higher compared with the control group after the treatment with 4-F-PVP at 10 mg/kg and 20 mg/kg. Moreover, mice treated with 20 mg/kg of 4-F-PVP traveled a longer distance than those treated with lower doses of 5 mg/kg and 10 mg/kg (Fig. 3d).

Similarly, vertical locomotor activity was significantly affected by the administration of 4-F-PVP, with the treatment (*F*_3,28_ = 15.85; *p* < 0.0001), time (*F*_11,308_ = 12.28; *p* < 0.0001), and treatment × time interaction (*F*_33,308_ = 7.727; *p* < 0.0001) being significant factors. Within 10-min bins, a number of rearings was significantly higher compared with the control in 90–100 min (5 mg/kg), 0–40 and 50–60 min (10 mg/kg) post-injection intervals, and for the entire 120 min of measurement after the highest dose of 20 mg/kg (Fig. 3e–g). A total number of rearings during 120 min was significantly higher after treatment with 20 mg/kg 4-F-PVP, both compared with the control group and 4-F-PVP at 5 mg/kg (Fig. 3h).

In mice treated with 4-MeO-PVP, horizontal locomotor activity was significantly affected by the treatment (*F*_3,28_ = 23.11; *p* < 0.0001), time (*F*_11,308_ = 23.96; *p* < 0.0001), and treatment × time interaction (*F*_33,308_ = 3.998; *p* < 0.0001). Within 10-min bins, horizontal activity was significantly increased compared with the control group after the treatment with 4-MeO-PVP during 20–30 min (5 mg/kg), 0–80 and 90–100 min (10 mg/kg) post-injection intervals, and through 120 min of the experiment at 20 mg/kg (Fig. 4a–c). A total distance traveled by mice during 120 min of analysis was significantly higher compared with the control group after the treatment with 4-MeO-PVP at 10 mg/kg and 20 mg/kg. Moreover, groups of mice treated with the two lower doses of 4-MeO-PVP (5 and 10 mg/kg) traveled significantly shorter distances compared with animals treated with 20 mg/kg 4-MeO-PVP (Fig. 4d).

Vertical locomotor activity in mice after the treatment with 4-MeO-PVP was significantly affected by the treatment (*F*_3,28_ = 25.57; *p* < 0.0001), time (*F*_11,308_ = 9.424; *p* < 0.0001), and treatment × time interaction (*F*_33,308_ = 3.240; *p* < 0.0001). Within 10-min bins, a significantly higher number of rearings compared with the control group was observed only after two higher doses of 4-MeO-PVP: 10 mg/kg (0–70 and 90–100 min post-injection) and 20 mg/kg (0–120 min post-injection) (Fig. 4e–g). A total number of rearings during 120 min was significantly higher in mice treated with 4-MeO-PVP at 20 mg/kg, compared with both the control group and mice treated with 5 mg/kg (Fig. 4h).

### Rotarod/Accelerod

During the last day of training (day 3), a time spent on the rotarod did not differ among groups, meaning that mice achieved a similar level of performance before the treatment with drugs (data not shown).

**Fig. 5:**
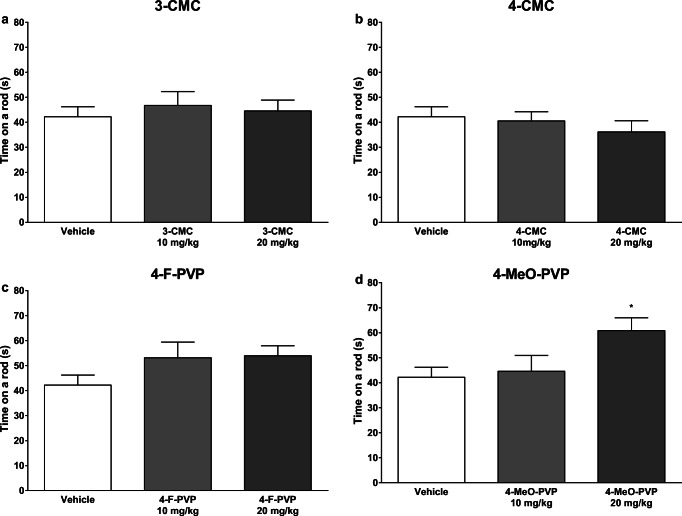
Effects of **a** 3-CMC (10, 20 mg/kg), **b** 4-CMC (10, 20 mg/kg), **c** 4-F-PVP (10, 20 mg/kg), and **d** 4-MeO-PVP (10, 20 mg/kg) on the performance of mice on the rotarod. Data presented as mean ± standard error of the mean (SEM) (*n* = 12—drug-treated groups or 14—control group). *p < 0.05 vs. control group

None of the compounds tested significantly reduced the time mice spent on the rotarod, meaning they do not produce an impairment of motor coordination (Fig. 5). However, in the case of pyrovalerone derivatives, a tendency to increase a latency to fall was observed. Mice treated with 4-F-PVP appeared to stay longer on the rod, but this difference did not reach a level of statistical significance. 4-MeO-PVP at the high dose of 20 mg/kg caused a significant extension of time the animals spent on the rotarod (Fig. 5d), an observation likely reflecting an increase of their forced locomotor activity (Giannotti et al. 2017).

### Cytotoxicity/Neurotoxicity

We have previously demonstrated that the exposure of SH-SY5Y neuroblastoma cells to 4-F-PVP and 4-MeO-PVP produces a moderate decline of their viability, measured as mitochondrial activity, in a time- and concentration-dependent manner. On the other hand, both compounds evoked only a slight damage to cell membranes at the highest concentration used (300 μM) after 48-h exposure (Wojcieszak et al. 2018a).

**Fig. 6:**
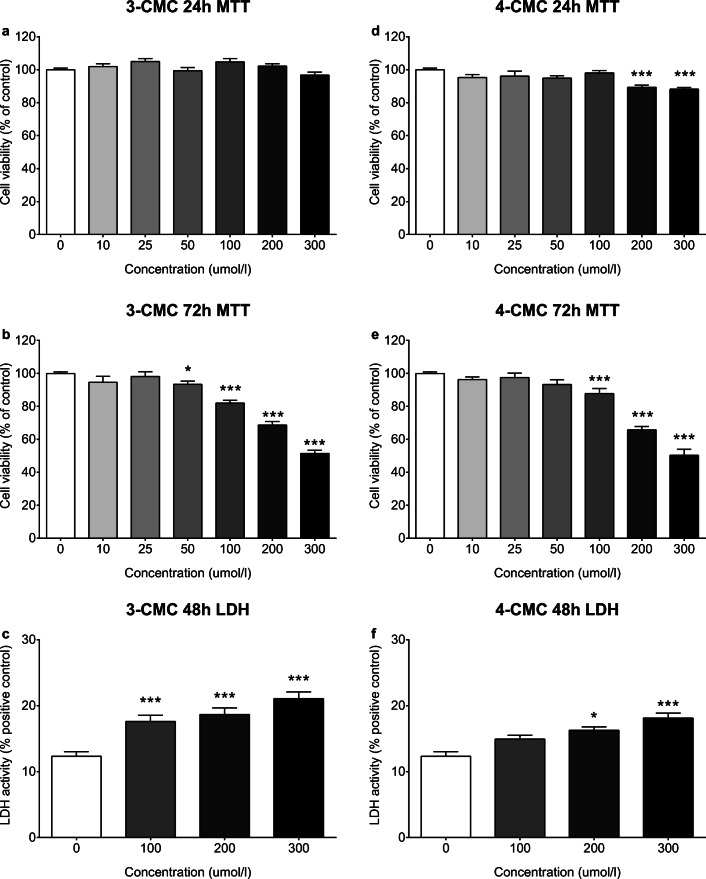
Cytotoxic effects of 3-CMC and 4-CMC against human SH-SY5Y neuroblastoma cells. Effects of **a**, **b** 3-CMC (10–300 μM) and **d**, **e** 4-CMC (10–300 μM) on the mitochondrial activity measured after 24-h or 72-h incubation with MTT test. Data presented as mean ± standard error of the mean (SEM) from at least 3 independent experiments and expressed as a percentage of control group considered 100% viable. Effects of **c** 3-CMC (100–300 μM) and **f** 4-CMC (100–300 μM) on the integrity of cell membranes after 48-h incubation measured with LDH test. Data presented as mean ± standard error of the mean (SEM) from at least 3 independent experiments and expressed as a percentage of positive control group considered 100% cytotoxicity. ***p < 0.001; **p < 0.01; *p < 0.05 vs. control group

Incubation of SH-SY5Y cells with 3-CMC for 24 h did not affect their viability (Fig. 6a). Extension of the exposition time to 72 h resulted in a significant decline of SH-SY5Y cell viability in the concentration range of 50–300 μM, with a maximal decrease by 49% of the control value at 300 μM (Fig. 6b). After 48-h incubation with 3-CMC (100, 200, and 300 μM), a significant damage to the cell membrane was observed, with a maximal effect of approx. 21% of positive control group at 300 μM (Fig. 6c).

Exposure of SH-SY5Y cells to 4-CMC for 24 h caused only a benign reduction of their viability at 200 μM and 300 μM, by 11% and 12%, respectively (Fig. 6d). Longer, 72-h incubation resulted in an intensification of cytotoxicity at 200 μM and 300 μM, and extension of cytotoxic concentration range to 100–300 μM. A maximal effect of 4-CMC after 72-h incubation, a decrease of cell viability by 50%, was observed at 300 μM (Fig. 6e). Incubation of SH-SY5Y cells with 200 and 300 μM 4-CMC for 48 h resulted in a significant damage to the cell membrane, with a maximal effect of approx. 18% of positive control group at 300 μM (Fig. 6f).

## Discussion

Assessment of the ability of drugs of abuse to increase spontaneous locomotor activity of rodents is an important tool to predict their abuse liability, as it is recognized that both reinforcing/rewarding properties are mediated by an increase of DA-ergic neurotransmission in the striatum, which is also manifested in the locomotor stimulation (Baumann et al. 2011; Blough et al. 2019; Bonano et al. 2015). Therefore, it is recommended that any CNS-active drug should be evaluated for its abuse potential both using in vitro and in vivo pharmacological models (FDA 2017; Mdege et al. 2017).

The present study examined effects of four synthetic cathinones from two chemical subgroups, halogenated methcathinone derivatives (3-CMC and 4-CMC) and α-pyrrolidinophenones (4-F-PVP and 4-MeO-PVP), on locomotor activity and performance on the rotarod in male C57BL/6J mice.

All test compounds increased spontaneous horizontal locomotor activity in the dose-dependent manner, and with similar potency. They produced significant locomotor stimulant effects at doses of 10 mg/kg and 20 mg/kg, while being inactive at 5 mg/kg. 4-F-PVP and 4-MeO-PVP also evoked the dose-dependent increases of vertical locomotor activity (rearing counts). On the other hand, 3-CMC was inactive at all tested doses, while the increase of vertical activity in mice treated with 4-CMC was significant only at the middle dose of 10 mg/kg. Recently, Gatch et al. (2019) demonstrated stimulation of horizontal locomotor activity in Swiss-Webster mice by 4-CMC (1–10 mg/kg). Reported effects were delayed, peaking 30–60 min after administration, and long lasting. In our study, the locomotor stimulant effect of 4-CMC was immediate at doses of 10 mg/kg and 20 mg/kg or delayed by 10 min at the 5 mg/kg dose; it lasted for a maximum 100 min. The above discrepancies could be related to different strains of mice (Swiss-Webster vs. C57BL/6J), and higher doses of the drug used in the current study, which favor reaching high CNS concentrations sooner. In addition, in our experimental protocol, mice freely explored the chamber for 30 min before administration of the drug. Due to this habituation, a very stable level of locomotor activity through the experiment was achieved in the control group, which in turn allowed detection of locomotor stimulation during the first minutes of analysis.

The results of the present study are in line with and expand our previous findings on properties of three α-pyrrolidinophenones (Wojcieszak et al. 2018b) and two methcathinones (Wojcieszak et al. 2019). Firstly, they demonstrate that besides a length of α-aliphatic side chain, also substituents in the phenyl ring affect the psychostimulant potency of α-pyrrolidinophenones. Previously, we found that only α-PVP, a compound which contains an unsubstituted phenyl ring and a side chain of 5 carbon atoms, used at 3 mg/kg significantly increased a total distance covered by mice during 120 min, while an elongation of the side chain by 2 or 3 carbon atoms led to compounds with a lower potency, active at the minimal dose of 10 mg/kg (Wojcieszak et al. 2018b). The current study demonstrates that a similar change in potency of drugs could be achieved by a substitution of the phenyl ring with the fluoride or methoxy group in *para* position. Chloride-substituted methcathinones, 3-CMC and 4-CMC, display a similar profile of action to previously evaluated methcathinone and 3-fluoromethcathinone, as all compounds used at 10 mg/kg effectively increased the total horizontal activity during 120 min of observation (Wojcieszak et al. 2019). Moreover, the results of the present study confirm our previous observations (Wojcieszak et al. 2018b, 2019) that α-pyrrolidinophenones, but not methcathinone and its phenyl-substituted derivatives, lead to consistent increases of vertical locomotor activity in mice.

**Fig. 7:**
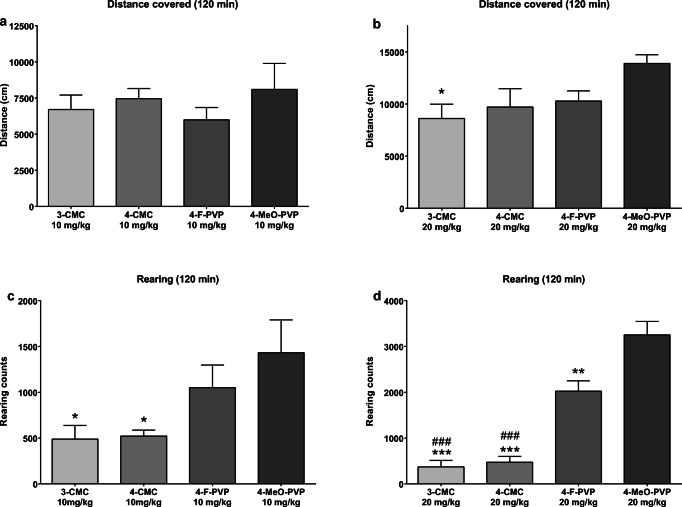
Comparison of the potency of 3-CMC, 4-CMC, 4-F-PVP, and 4-MeO-PVP to stimulate the spontaneous horizontal activity of mice at 10 mg/kg (**a**) and 20 mg/kg (**b**) and the spontaneous vertical locomotor activity of mice at 10 mg/kg (**c**) and 20 mg/kg (**d**). Figure constructed with data presented in Figs. 1, 2, 3, and 4. Data presented as mean ± standard error of the mean (SEM) (*n* = 8). ***p < 0.001; **p < 0.01; *p < 0.05 vs. 4-MeO-PVP group; ###*p* < 0.001 vs. 4-F-PVP group

Stimulation of horizontal locomotor activity had a similar magnitude after the treatment with either of the four tested cathinones at 10 mg/kg, as no significant differences among groups were observed (Fig. 7a). After the treatment with the higher dose (20 mg/kg), the only significant difference was between 3-CMC and 4-MeO-PVP (Fig. 7b). On the other hand, changes in the vertical locomotor activity seem to be more specific to the drugs’ structure and mechanism of action. Thus, chloromethcathinones, which act as DA and 5-HT reuptake inhibitors and releasers, equipotent at DAT and SERT (4-CMC) or with a slight preference for DAT (3-CMC, DAT selectivity 8.8), at the dose of 10 mg/kg produced significantly lower vertical locomotor stimulation compared with 4-MeO-PVP (a potent and highly DAT-selective reuptake inhibitor) (Fig. 7c), and at 20 mg/kg were markedly less active than either 4-F-PVP or 4-MeO-PVP (Fig. 7d) (Blough et al. 2019; Bonano et al. 2015; Eshleman et al. 2017).

Therefore, although it is widely accepted that in rats compounds with high DAT selectivity are potent forward locomotor stimulators (Baumann et al. 2011; Blough et al. 2019), our data suggest that vertical locomotor activity in mice could be more associated with a monoaminergic profile of synthetic cathinones than the horizontal one. We admit that this assumption has a limitation resulting from a lack of published data on a pharmacological profile of 4-F-PVP. However, based on the available pharmacological data, it can be generalized that α-pyrrolidinophenones are highly potent reuptake inhibitors with the significant preference for DAT over SERT (Eshleman et al. 2017; Rickli et al. 2015). On the other hand, based on our previous report (Wojcieszak et al. 2018b), the potency of a drug to increase horizontal locomotor activity may serve as an index of its ability to increase the extracellular DA levels in the striatum, as in contrast to the similar activity observed in vitro (Eshleman et al. 2017), experiments on animals revealed that α-PVP produced stronger locomotor stimulation than PV8, which was reflected in the more prominent increase of extracellular DA levels in vivo after treatment with α-PVP.

In the second part of our study, we examined effects of synthetic cathinones on the performance of mice in the rotarod test. In general, the rotarod test is used either to estimate impairment of motor coordination and balance resulting from neurotoxicity, which would be detected if the latency to fall was decreased (Deacon 2013; Marusich et al. 2012; Shiotsuki et al. 2010), or as a measure of stimulation of forced locomotor activity, where an increase of time the animal remains on the rod is expected (Giannotti et al. 2017). None of the tested compounds (3-CMC, 4-CMC, 4-F-PVP, and 4-MeO-PVP) reduced time the mice remained on the rotarod. On the other hand, 4-MeO-PVP at the high dose of 20 mg/kg significantly increased this parameter. Up to date, an assessment of effects of different synthetic cathinones on the performance of mice on the rotarod apparatus has been done twice, however, using different protocols (Giannotti et al. 2017; Marusich et al. 2012). Using rotarod revolving at a constant, low speed of 10 rpm, Marusich et al. (2012) did not observe significant changes in the performance of mice treated with 4-FMC, 3,4-MDPV, mephedrone, and methedrone, while 3-FMC and methylone at 56 mg/kg significantly reduced time spent on the rod, an effect likely resulting from ataxia produced by the high dose of the drugs. Using accelerating rotarod, Giannotti et al. (2017) demonstrated that two pyrrolidine-containing cathinones, 3,4-MDPV and α-PVP, markedly improved the performance of mice. They postulated that the observed improvement in the mouse behavior is due to an increased forced locomotor activity, and involves stimulation of DA-ergic neurotransmission in the striatum. This hypothesis is in accordance with our present data. Both in our study and experiments done by Giannotti and coworkers, all cathinones that increased time spent by mice on the accelerating rod belong to the group of α-pyrrolidinopentiophenones, and have a very strong preference for DAT inhibition over SERT (Eshleman et al. 2017; Rickli et al. 2015). In addition, α-PVP, a more potent compound than 4-MeO-PVP in terms of inhibiting DAT and selectivity for DAT over SERT (Eshleman et al. 2017), increased the performance of mice on the rotarod starting at 10 mg/kg (Giannotti et al. 2017), while 4-MeO-PVP was effective at 20 mg/kg (present data). This observation is supported by the fact that α-PVP is more potent at inhibiting DAT and more selective for DAT over SERT compared with 4-MeO-PVP (Eshleman et al. 2017), which is also consistent with the fact that α-PVP stimulated both horizontal and vertical locomotor activities in mice at lower doses than 4-MeO-PVP (Wojcieszak et al. 2018b).

Based on the aforementioned observations, it could be hypothesized that both the increase of vertical locomotor activity and improvement in performance on the accelerating rotarod is characteristic for compounds with high DAT/SERT selectivity, and depends on their high potency to inhibit DAT. Therefore, an assessment of these effects could be applied as a behavioral test to confirm the pharmacological profile of synthetic cathinones obtained from in vitro studies.

Despite the growing popularity of chloromethcathinones on the recreational drugs’ market and documented cases of acute intoxication in humans, little is known on their cytotoxic activity (Adamowicz et al. 2020; Grifell et al. 2017; Odoardi et al. 2016; Taschwer et al. 2014; Tomczak et al. 2018). The present study demonstrates that 3-CMC does not produce significant cytotoxicity against SH-SY5Y neuroblastoma cells after 24-h incubation, while 4-CMC slightly, but statistically significantly decreases cell viability, measured as mitochondrial function, at 200 and 300 μM. As a result of prolonged exposure time to 72 h, both compounds show considerable cytotoxicity, starting at 50 and 100 μM, respectively, and reaching a maximal effect of approx. 50% of cell death. Consistently, after 48-h incubation, the cell membrane damage is observed after the treatment with both 3-CMC and 4-CMC, while 3-CMC exerts significant effects at a lower concentration. Although a direct mechanism of cytotoxicity was not evaluated, based on literature data, it seems likely that generation of toxic metabolites plays an important role. This hypothesis is supported by a recent study showing that 4-CMC was the least stable of studied cathinones, and its half-life at a room temperature was < 1 day in blood and approx. 10 days in urine, suggesting a rapid enzymatic breakdown (Adamowicz and Malczyk 2019). Importantly, a similar case, where one of the major metabolites was found to be more cytotoxic than the parent compound, was reported for 3,4-MDPV (Coccini et al. 2019; Wojcieszak et al. 2016). The delay of emergence of cytotoxic effects of both chloromethcathinones distinguishes them from previously reported properties of 4-MeO-PVP and 4-F-PVP (Wojcieszak et al. 2018a). Although the maximal effect on the mitochondrial activity after 72-h incubation and the damage to cell membranes after 48 h are of comparable magnitude, *para*-substituted α-pyrrolidinopentiophenones exert more pronounced cytotoxicity against SH-SY5Y cells after shorter, 24-h incubation, compared with 3-CMC and 4-CMC (Wojcieszak et al. 2018a).

A few documented reports on cytotoxicity of chloromethcathinones have been published. Fluoride and chloride share many chemical properties, as they are highly reactive and electrophilic halogens with a low molecular mass. As a result, halogenation using fluoride or chloride is a common modification done in order to obtain new designer drugs with similar properties. Pharmacological and toxicological properties of chloro- and fluoro-derivatives are often compared (Luethi et al. 2019; Suyama et al. 2016). 4-Chloromethcathinone at millimolar concentrations has been found to diminish the viability of Hep G2 cells, widely accepted as a model of hepatotoxicity, with greater potency compared with 4-fluoromethcathinone (4-FMC) (Luethi et al. 2019). Similarly, 4-FMC exerted cytotoxicity against differentiated SH-SY5Y cells after 24-h exposure, starting at 500 μM (Soares et al. 2019), which is higher than toxic concentrations of 4-CMC demonstrated by us. This observation confirms findings of Luethi et al. (2019) that toxicity of *para*-substituted methcathinone analogs can be ordered as follows: chloride > fluoride > hydrogen. Although one may dispute that different potencies of 4-CMC and 4-FMC could result from using differentiated SH-SY5Y cells by Soares et al. (2019) and undifferentiated cells in the current study, it should be noted that in both cases cytotoxic concentrations of the drugs are substantially higher than their IC_50_ values for monoamine transporters (Luethi et al. 2019), indicating that the mechanism of cytotoxicity most likely does not involve an interaction of drugs with DAT, NET, or SERT. Interestingly, the discussed difference in cytotoxic properties of *para*-halogenated methcathinones points to the necessity to characterize and assess toxic effects of major 4-CMC and 4-FMC metabolites, as according to Adamowicz and Malczyk (2019), 4-FMC has two times longer half-life in the blood than 4-CMC, suggesting that potentially toxic metabolites of 4-CMC can be generated faster. The deleterious effects of halogenated methcathinones are not limited to the *para*-derivatives, as we currently demonstrate neurotoxic properties of *meta*-substituted 3-CMC, and there are reports on the cytotoxic effect of 3-FMC against mouse hippocampal neuronal HT22 cells (Siedlecka-Kroplewska et al. 2014, 2018). Although 3-FMC caused inhibition of cell growth and induced cell death at concentrations of 1 mM and higher, the comparison of potency with 3-CMC is not possible because of the use of cells of different origin.

## Conclusions

Taken together, the current study demonstrates that two chloromethcathinones, namely 3-CMC and 4-CMC, and two *para*-substituted α-pyrrolidinopentiophenones, 4-MeO-PVP and 4-F-PVP, dose-dependently stimulate spontaneous horizontal locomotor activity in mice, while only pyrovalerones produce dose-dependent elevation of vertical locomotor activity. The position of the chloride substituent in the phenyl ring of chloromethcathinones does not affect their psychostimulant potency, while at the highest test dose (20 mg/kg), 4-MeO-PVP produces stronger stimulation of vertical locomotor activity than 4-F-PVP and both CMCs. In accordance with this, only 4-MeO-PVP increases the performance of mice on the accelerating rotarod at 20 mg/kg. None of test drugs decreases the latency to fall, pointing to the lack of considerable deficits in motor coordination of mice after acute exposition. As α-pyrrolidinophenones are demonstrated to be highly potent and selective DA uptake inhibitors, in contrast to chloromethcathinones being non-selective DA/5-HT releasers, the ability to increase vertical locomotor activity and performance on the rotarod in mice seems to be a behavioral indicator of the profile of action of synthetic cathinones. Additionally, the current study demonstrates that both 3-CMC and 4-CMC are endowed with cytotoxic activity against SH-SY5Y cells, which emerges and intensifies after prolonged incubation, suggesting the indirect mechanism of action.

## References

[CR1] Adamowicz P, Jurczyk A, Gil D, Szustowski S (2020). A case of intoxication with a new cathinone derivative α-PiHP—a presentation of concentrations in biological specimens. Leg Med (Tokyo).

[CR2] Adamowicz P, Malczyk A (2019). Stability of synthetic cathinones in blood and urine. Forensic Sci Int.

[CR3] Baumann MH, Clark RD, Woolverton WL, Wee S, Blough BE, Rothman RB (2011). In vivo effects of amphetamine analogs reveal evidence for serotonergic inhibition of mesolimbic dopamine transmission in the rat. J Pharmacol Exp Ther.

[CR4] Białas T, Barczuk-Martuszewska K, Posobkiewicz M, Kucharska I, Klimberg A (2017). New drugs in Poland—latest trends according to State Sanitary Inspection data of 2015-2016. Hygeia Public Health.

[CR5] Blough BE, Decker AM, Landavazo A, Namjoshi OA, Partilla JS, Baumann MH, Rothman RB (2019). The dopamine, serotonin and norepinephrine releasing activities of a series of methcathinone analogs in male rat brain synaptosomes. Psychopharmacology.

[CR6] Bonano JS, Banks ML, Kolanos R, Sakloth F, Barnier ML, Glennon RA, Cozzi NV, Partilla JS, Baumann MH, Negus SS (2015). Quantitative structure-activity relationship analysis of the pharmacology of para-substituted methcathinone analogues. Br J Pharmacol.

[CR7] Coccini T, Vecchio S, Crevani M, De Simone U (2019). Cytotoxic effects of 3,4-catechol-PV (one major MDPV metabolite) on human dopaminergic SH-SY5Y cells. Neurotox Res.

[CR8] Coppola M, Mondola R (2012). Synthetic cathinones: chemistry, pharmacology and toxicology of a new class of designer drugs of abuse marketed as “bath salts” or “plant food”. Toxicol Lett.

[CR9] Deacon RMJ (2013). Measuring motor coordination in mice. J Vis Exp.

[CR10] den Hollander B, Sundström M, Pelander A, Siltanen A, Ojanperä I, Mervaala E, Korpi ER, Kankuri E (2015). Mitochondrial respiratory dysfunction of substituted cathinones to methylbenzamides in SH-SY5Y cells. Sci Rep.

[CR11] Ellefsen KN, Wohlfarth A, Swortwood MJ, Diao X, Concheiro M, Huestis MA (2016). 4-Methoxy-α-PVP: in silico prediction, metabolic stability, and metabolite identification by human hepatocyte incubation and high-resolution mass spectrometry. Forensic Toxicol.

[CR12] EMCDDA (2017) European drug report. Trends and developments http://wwwemcddaeuropaeu/system/files/publications/4541/TDAT17001ENNpdf Accessed 9 July 2019

[CR13] EMCDDA (2018) European drug report. Trends and developments http://wwwemcddaeuropaeu/system/files/publications/8585/20181816_TDAT18001ENN_PDFpdf Accessed 9 July 2019

[CR14] EMCDDA (2019) European drug report. Trends and developments http://wwwemcddaeuropaeu/system/files/publications/11364/20191724_TDAT19001ENN_PDFpdf Accessed 9 July 2019

[CR15] Eshleman AJ, Wolfrum KM, Hatfield MG, Johnson RA, Murphy KV, Janowsky A (2013). Substituted methcathinones differ in transporter and receptor interactions. Biochem Pharmacol.

[CR16] Eshleman AJ, Wolfrum KM, Reed JF, Kim SO, Swanson T, Johnson RA, Janowsky A (2017). Structure-activity relationships of substituted cathinones, with transporter binding, uptake, and release. J Pharmacol Exp Ther.

[CR17] Feng LY, Battulga A, Han E, Chung H, Li JH (2017). New psychoactive substances of natural origin: a brief review. J Food Drug Anal.

[CR18] Food and Drug Administration (2017) Assessment of abuse potential of drugs. Guidance for Industry https://wwwfdagov/media/116739/download Accessed 27 January 2020

[CR19] Gatch MB, Dolan SB, Forster MJ (2019). Locomotor activity and discriminative stimulus effects of five novel synthetic cathinone analogs in mice and rats. Drug Alcohol Depend.

[CR20] Giannotti G, Canazza I, Caffino L, Bilel S, Ossato A, Fumagalli F, Marti M (2017). The cathinones MDPV and α-PVP elicit different behavioral and molecular effects following acute exposure. Neurotox Res.

[CR21] Grifell M, Ventura M, Carbón X, Quintana P, Galindo L, Palma Á, Fornis I, Gil C, Farre M, Torrens M (2017) Patterns of use and toxicity of new para-halogenated substituted cathinones: 4-CMC (clephedrone), 4-CEC (4-chloroethcatinone) and 4-BMC (brephedrone). Hum Psychopharmacol 32(3). 10.1002/hup.2621 10.1002/hup.2621 28657185

[CR22] Luethi D, Walter M, Zhou X, Rudin D, Krähenbühl S, Liechti ME (2019). Para-halogenation affects monoamine transporter inhibition properties and hepatocellular toxicity of amphetamines and methcathinones. Front Pharmacol.

[CR23] Marusich JA, Grant KR, Blough BE, Wiley JL (2012). Effects of synthetic cathinones contained in “bath salts” on motor behavior and a functional observational battery in mice. Neurotoxicology.

[CR24] Matsunaga T, Morikawa Y, Kamata K, Shibata A, Miyazano H (2017). α-Pyrrolidinononanophenone provokes apoptosis of neuronal cells through alterations in antioxidant properties. Toxicology.

[CR25] Mdege ND, Meader N, Lloyd C, Parrott S, McCambridge J (2017) The Novel Psychoactive Substances in the UK Project: empirical and conceptual review work to produce research recommendations. Chapter 5 research recommendations. Public Health Res 5(4):61. 10.3310/phr05040 28678463

[CR26] Odoardi S, Romolo FS, Strano-Rossi S (2016). A snapshot on NPS in Italy: distribution of drugs in seized materials analysed in an Italian forensic laboratory in the period 2013-2015. Forensic Sci Int.

[CR27] Rickli A, Hoener MC, Liechti ME (2015). Monoamine transporter and receptor interaction profiles of novel psychoactive substances: para-halogenated amphetamines and pyrovalerone cathinones. Eur Neuropsychopharmacol.

[CR28] Shiotsuki H, Yoshimi K, Shimo Y, Funayama M, Takamatsu Y, Ikeda K, Takahashi R, Kitazawa S, Hattori N (2010). A rotarod test for evaluation of motor skill learning. J Neurosci Methods.

[CR29] Siedlecka-Kroplewska K, Szczerba A, Lipinska A, Slebioda T, Kmiec Z (2014). 3-Fluoromethcathinone, a structural analog of mephedrone, inhibits growth and induces cell cycle arrest in HT22 mouse hippocampal cells. J Physiol Pharmacol.

[CR30] Siedlecka-Kroplewska K, Wrońska A, Stasiłojć G, Kmieć Z (2018). The designer drug 3-fluoromethcathinone induces oxidative stress and activates autophagy in HT22 neuronal cells. Neurotox Res.

[CR31] Simmler LD, Buser TA, Donzelli M, Schramm Y, Dieu L-H, Huwyler J, Chaboz S, Hoener MC, Liechti ME (2013). Pharmacological characterization of designer cathinones in vitro. Br J Pharmacol.

[CR32] Simmons SJ, Leyrer-Jackson JM, Oliver CF, Hicks C, Muschamp JW, Rawls SM, Olive MF (2018). DARK classics in chemical neuroscience: cathinone-derived psychostimulants. ACS Chem Neurosci.

[CR33] Soares J, Costa VM, Gaspar H, Santos S, de Lourdes BM et al (2019) Structure-cytotoxicity relationship profile of 13 synthetic cathinones in differentiated human SH-SY5Y neuronal cells. Neurotoxicology 75:158–17310.1016/j.neuro.2019.08.009 10.1016/j.neuro.2019.08.009 31473217

[CR34] Suyama JA, Sakloth F, Kolanos R, Glennon RA, Lazenka MF, Negus SS, Banks ML (2016). Abuse-related neurochemical effects of para-substituted methcathinone analogs in rats: microdialysis studies of nucleus accumbens dopamine and serotonin. J Pharmacol Exp Ther.

[CR35] Taschwer M, Weiß JA, Kunert O, Schmid MG (2014). Analysis and characterization of the novel psychoactive drug 4-chloromethcathinone (clephedrone). Forensic Sci Int.

[CR36] Tomczak E, Woźniak MK, Kata M, Wiergowski M, Szpiech B, Biziuk M (2018) Blood concentrations of a new psychoactive substance 4-chloromethcathinone (4-CMC) determined in 15 forensic cases. Forensic Toxicol 36(2):476–485. 10.1007/s11419-018-0427-8 10.1007/s11419-018-0427-8 PMC6002423 29963211

[CR37] Valente MJ, de Lourdes BM, Fernandes E, Carvalho F, Guedes de Pinho P (2017). Neurotoxicity of β-keto amphetamines: deathly mechanisms elicited by methylone and MDPV in human dopaminergic SH-SY5Y cells. ACS Chem Neurosci.

[CR38] Wiergowski M, Aszyk J, Kaliszan M, Wilczewska K, Anand JS, Kot-Wasik A, Jankowski Z (2017). Identification of novel psychoactive substances 25B-NBOMe and 4-CMC in biological material using HPLC-Q-TOF-MS and their quantification in blood using UPLC-MS/MS in case of severe intoxications. J Chromatogr B.

[CR39] Wojcieszak J, Andrzejczak D, Kedzierska M, Milowska K, Zawilska JB (2018). Cytotoxicity of α-pyrrolidinophenones: an impact of α-aliphatic side-chain length and changes in the plasma membrane fluidity. Neurotox Res.

[CR40] Wojcieszak J, Andrzejczak D, Wojtas A, Gołembiowska K, Zawilska JB (2018). Effects of the new generation α-pyrrolidinophenones on spontaneous locomotor activities in mice, and on extracellular dopamine and serotonin levels in mouse striatum. Forensic Toxicol.

[CR41] Wojcieszak J, Andrzejczak D, Wojtas A, Gołembiowska K, Zawilska JB (2019). Methcathinone and 3-fluoromethcathinone stimulate spontaneous horizontal locomotor activity in mice and elevate extracellular dopamine and serotonin levels in the mouse striatum. Neurotox Res.

[CR42] Wojcieszak J, Andrzejczak D, Woldan-Tambor A, Zawilska JB (2016). Cytotoxic activity of pyrovalerone derivatives, an emerging group of psychostimulant designer cathinones. Neurotox Res.

[CR43] Zawilska JB, Wojcieszak J (2013). Designer cathinones—an emerging class of novel recreational drugs. Forensic Sci Int.

[CR44] Zawilska JB, Wojcieszak J (2017). α-Pyrrolidinophenones: a new wave of designer cathinones. Forensic Toxicol.

